# The Aftermath: Post-pandemic Psychiatric Implications of the COVID-19 Pandemic, a South Korean Perspective

**DOI:** 10.3389/fpsyt.2021.671722

**Published:** 2021-10-21

**Authors:** Sooyeon Min, Yun Ha Jeong, Jeongyeon Kim, Ja Wook Koo, Yong Min Ahn

**Affiliations:** ^1^Department of Neuropsychiatry, Seoul National University Hospital, Seoul, South Korea; ^2^Research Headquarters, Korea Brain Research Institute, Daegu, South Korea; ^3^Department of Brain and Cognitive Sciences, Daegu Gyeongbuk Institute of Science and Technology, Daegu, South Korea; ^4^Institute of Human Behavioral Medicine, Seoul National University College of Medicine, Seoul, South Korea

**Keywords:** COVID-19, post-pandemic, mental health, general populations, honeymoon phase, new normal, South Korea

## Abstract

The coronavirus disease 2019 (COVID-19) pandemic has disrupted our everyday life. Along with the fear of getting infected or of having loved ones infected, the lifestyle changes and the socioeconomic consequences of the pandemic have profound impact on mental health of the general population. While numerous studies on immediate psychological responses to COVID-19 are being published, there is a lack of discussion on its possible long-term sequelae. In this study, we systematically reviewed and meta-analyzed longitudinal studies that examined mental health of the general population prior to and during the pandemic. Furthermore, we explored the long-term psychiatric implications of the pandemic with data from South Korea. Our analysis showed that the number of suicidal deaths during the pandemic was lower than the previous years in many countries, which is in contrast with the increased depression, anxiety, and psychological distress in the general population in South Korea as well as in other countries. To explain this phenomenon, we propose a possibility of delayed impacts. The post-traumatic stress, long-term consequences of social restrictions, and maladaptive response to the “new normal” are discussed in the paper. COVID-19 being an unprecedented global crisis, more research and international collaboration are needed to understand, to treat, and to prevent its long-term effects on our mental health.

## Introduction

The coronavirus disease 2019 (COVID-19) evolved into a worldwide pandemic infecting more than 220 million individuals and claiming 4.5 million lives worldwide as of September 9, 2021 (1). The pandemic has brought considerable disruption to the way most people live, work, study, and access health care. These changes and their socioeconomic consequences, along with the fear of getting infected or of having loved ones infected have a profound impact on the mental health of the general population. Previous research about past pandemics, such as the 2003 outbreak of Severe Acute Respiratory Syndrome (SARS), has shown higher rates of depression, anxiety, insomnia, and post-traumatic stress in the general population (2, 3) as well as in people with pre-existing mental illness, health care workers, and survivors of severe cases of the disease (4, 5). As one of the first countries to be affected by COVID-19 (6), South Korea's early implementation of testing, contact tracing, and social distancing has been recognized worldwide as successful measures that brought the virus under control (7). However, despite the well-recognized efforts, the country has recently faced at fourth wave of the viral epidemic with its peaks reaching over 2,000 newly confirmed cases per day, far above previous outbreaks.

There is a growing number of reports about mental health impacts of the COVID-19 outbreak (8–10), as well as the physical health consequences of COVID-19, in many countries such as China (11–15), Italy (16, 17), India (18, 19), Mexico (20), the United Kingdom (21, 22), the USA (23), and Spain (24, 25). In marked contrast to the rapidly growing literature “during” the COVID-19 pandemic, there is lack of published discussion on the mental health of the general population “after” the pandemic (26–28). Here, we aim to raise public awareness of putative prolonged impacts of the COVID-19 pandemic on mental health through a review of current knowledge on the impact of the pandemic on the mental health of the general population, and of data from South Korea (29, 30). The hypothesis of the present study is that COVID-19 has detrimental effects on mental health and suicide rates. We furthermore discuss the maladaptive response to a “new normal” lifestyle triggered by the COVID-19 pandemic.

## Methods

### Study Selection and Data Extraction

A systematic search was conducted for longitudinal studies that measured changes in mental health of the general population since the COVID-19 pandemic that were published from January 1, 2020 to July 12, 2021. Electronic searches using subject headings (i.e., MeSH terms) and free-text keywords (an example shown in Supplementary Table 1) involved five electronic databases: PubMed, Scopus, Web of Science, APA PsychInfo, and CINAHL. According to the indices of each database, key search terms used for mental health included “mental health,” “mental illness,” “mental disorder,” “depression,” “anxiety,” “stress,” “post-traumatic stress disorder,” and “suicide.” Key search terms used for COVID-19 included “coronavirus disease 2019,” “novel coronavirus,” and “SARS-CoV-2.”

Authors independently screened the titles and abstracts, and reviewed the full text articles to select studies meeting the following criteria: studies (a) with longitudinal designs; (b) that assessed psychological symptoms before and during the COVID-19 pandemic using the same measurement tools; (c) that are validated and standardized. The study selection process is shown in Supplementary Figure 1 and the characteristics of included studies are shown in Supplementary Table 2. For more details about meta-analysis, see Supplementary Materials.

## Results

Three hundred sixty-one citations were retrieved from the electronic databases and 10 citations were identified through manual search. Three hundred thirty-one studies remained after removing duplicates and 165 after screening titles and abstracts. We assessed full-text articles and selected 22 articles for systematic review. Among the 22 articles, seven studies were excluded from the quantitative synthesis as their reported outcome values were not comparable to that of other studies; for instance, Ramiz et al. was excluded from the final meta-analysis as it only reported the prevalence of anxiety with a cut-off GAD-7 > 4 (mild or greater anxiety) (31), while most of the studies reported prevalence of anxiety of clinically significant level, equivalent of GAD-7 ≥ 10. All included studies were repeated cross-sectional studies, while five of them were conducted in the USA, four in the UK, two in the Netherlands, one in Japan, and others in other European countries.

### Depression

**Figure 1:**
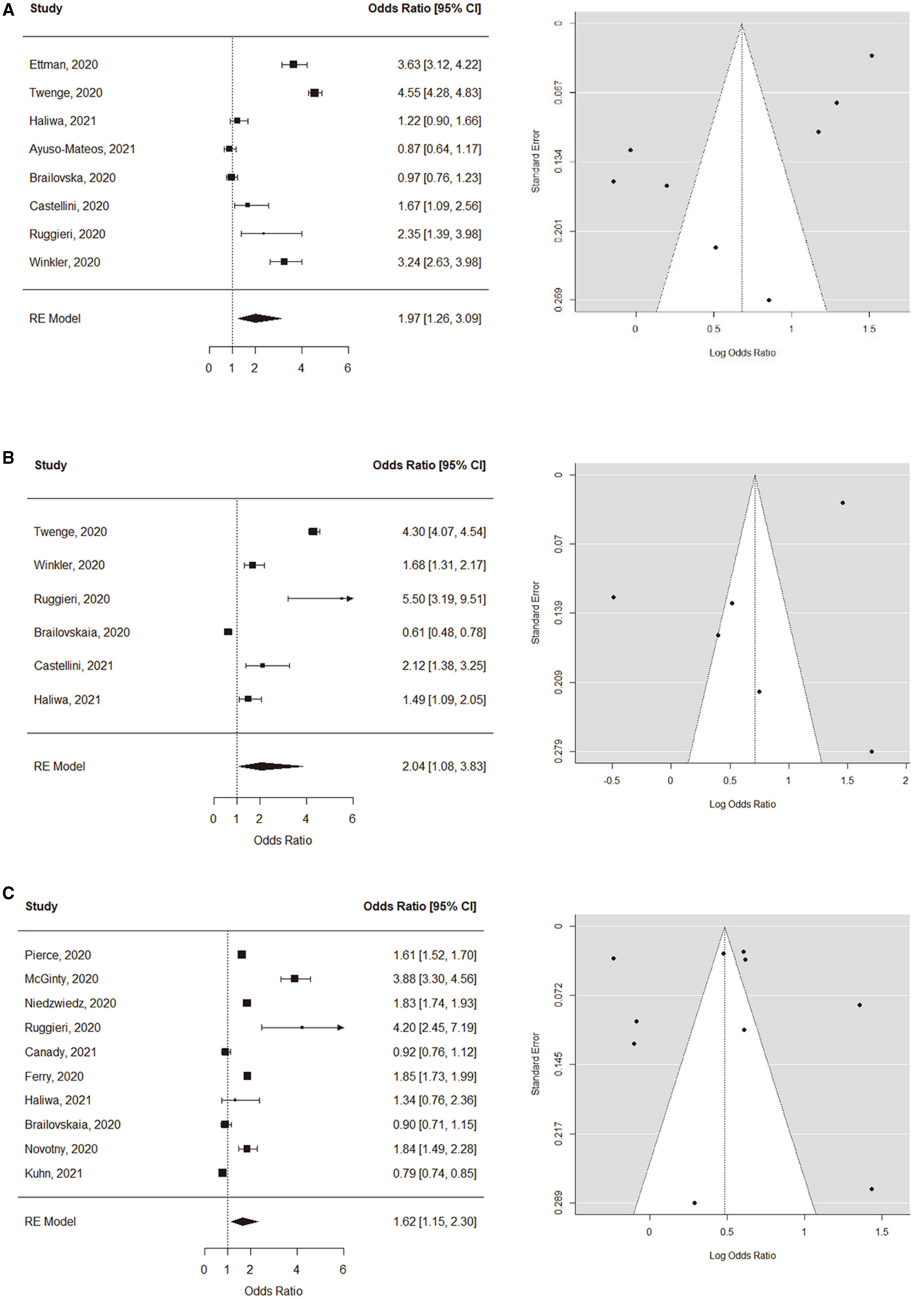
Forest and funnel plots of symptom level comparisons. **(A)** Depression, **(B)** Anxiety, and **(C)** Psychological distress and stress.

Ten studies quantified and compared the level of depression in the general population before vs. during the pandemic. The studies measured the level of depressive symptoms using scales – such as the Patient Health Questionnaire (PHQ-9, PHQ-8, or PHQ-2), the Depression, Anxiety and Stress Scale (DASS-21), or the Brief Symptom Inventory (BSI) – or estimated the prevalence of major depressive episode using the Mini International Neuropsychiatric Interview (M.I.N.I.). The overall pooled odds ratio was 1.97 (95% CI: 1.26–3.09, see Figure 1A), showing a significant increase in depression since the pandemic. A high degree of heterogeneity was found across the studies (*I*^2^ = 97.6%, Q = 319.3, *p* < 0.001). We performed a sensitivity analysis to explore the impact of measurement tool by limiting the analysis to the studies using the PHQ and found consistent results: the overall pooled odds ratio was 2.75 (95% CI: 1.26–6.04, see Supplementary Figure 2A). (32, 33) reported no change or decrease in the prevalence of high Anxiety and Depression Symptoms (ADS) levels in the Dutch population-based longitudinal studies, which were measured using the 5-item Mental Health Index (MHI-5). The two studies did not report any measure specific to depression, thus were excluded from the meta-analysis. In an exploratory analysis with a cut-off PHQ-9/-8 ≥ 5 (mild or greater depression), there was no significant change in the prevalence after the pandemic (Supplementary Figure 2B).

### Anxiety

Six studies were included in the meta-analysis for the change in the prevalence of clinically significant level of anxiety before vs. during the pandemic, defined as GAD-7 ≥ 10 or GAD-2 ≥ 3. The overall pooled odds ratio was 2.04 (95% CI: 1.08–3.83, see Figure 1B), suggesting a significant increase since the pandemic. A high degree of heterogeneity was found (*I*^2^ = 97.7%, Q = 317.6, *p* < 0.001).

### Psychological Distress/Stress

Ten studies compared the prevalence of clinically significant level of psychological distress, defined as the 12-item General Health Questionnaire (GHQ-12) ≥ 4 or the 6-item Kessler Psychological Distress Scale (K6) ≥ 13. The overall pooled odds ratio was 1.62 (95% CI: 1.15–2.30, see Figure 1C). The heterogeneity among studies was high (*I*^2^ = 99.2%, Q = 648.0, *p* < 0.001).

### Publication Bias Assessment

Funnel plots were first created for visual inspection to determine whether the included studies showed publication bias (see Figure 1). Egger's regression tests were additionally performed, and no significant publication bias was found in the studies regarding depression (β = 1.22, CI: 0.29–2.16, *p* = 0.20), anxiety (β = 10.46, CI: −1.08 to 2.00, *p* = 0.71), or psychological distress (β = 0.33, CI: −0.22 to 0.88, *p* = 0.47).

## Discussion

To our knowledge, this is the first systematic review and meta-analysis of longitudinal studies that examined the change in prevalence of psychiatric symptoms in the general population since the COVID-19 pandemic. According to our analysis, there was a significant increase in depression (OR = 1.97), anxiety (OR = 2.04), and psychological distress (OR = 1.62) in the general population. Since the beginning of the COVID-19 pandemic, experts warned of its possible destructive impact on the mental health of the general population. Although substantial heterogeneity exists between the studies on mental health during the pandemic, we can conclude that the virus outbreak has led to significant consequences in mental health in the affected populations.

### Suicidal Ideation and Suicide Rates

A number of psychiatrists pointed out the possibility of an increase in suicide rates (34, 35), as the rise in psychiatric symptoms may remain untreated and be accompanied by socioeconomic burden. In contrast to such concerns, and to the results of our meta-analysis that showed increased depression, anxiety, and psychological distress, the suicidal ideation and suicide rates have not shown a remarkable rise since the pandemic.

A comprehensive review on the suicide behaviors during the pandemic reported no increase during the pandemic above pre-pandemic levels (36, 37). A couple of more recent studies, which investigated the impact of the COVID-19 pandemic on suicidal ideation in the general population though a longitudinal survey, in which measurements occurred in both pre-pandemic and pandemic periods, also showed no change or a small decrease (38, 39). Similarly, Google searching for suicide-related queries in Italy, Spain, the USA, the UK, and worldwide significantly declined after the pandemic declaration (40–42), although they increased again since the announcement of lockdown in each country (41).

A recent study that collected and analyzed real-time suicide data from 21 countries concluded that the actual number of suicides in the context of the COVID-19 pandemic in many countries remained unchanged or declined in the early phase of the pandemic compared with the anticipated levels based on the pre-pandemic period (43). The case of Japan, another country to among one of the first to be affected by the pandemic, is interesting. During the initial 5 months following the first wave of the outbreak (February to June 2020), the number of suicides decreased by 14% as in other countries, while after the second wave (July to October 2020), suicide rates increased, notably in greater magnitude among women, children, and adolescents (44, 45).

### National Survey on the Mental Health in South Korea

The results of the most recent national survey on the mental health of South Korean population, fourth since March 2020, have recently been announced by the Korean Society for Traumatic Stress Studies (KSTSS) (29). The surveys reported that the level of anxiety – i.e., population at risk of clinically significant level of anxiety, defined as a General Anxiety Disorder-7 (GAD-7) score > 10 – increased in reaction to the outbreak and decreased when the virus seemed to be contained. However, the level of depression – i.e., population at risk of clinically significant level of depression, defined as a Patient Health Questionnaire-9 (PHQ-9) score > 10 – continued to increase from 17.5 to 20.0% from March 2020 to January 2021. This is a remarkable escalation from the rate of 3.8% reported by the Community Health Survey done in 2018 (29). The number of people with suicidal idea also increased from 9.7 to 13.4% from March 2020 to January 2021 (29). This suggests that while people suffer with anxiety in reaction to the severity of the outbreaks, they continue to accumulate symptoms of depression as the epidemic continues. Female and younger age (19–29 years old) groups were associated with a greater risk of depression (29). These findings are consistent with some systematic reviews on the subject (46–48).

**Figure 2:**
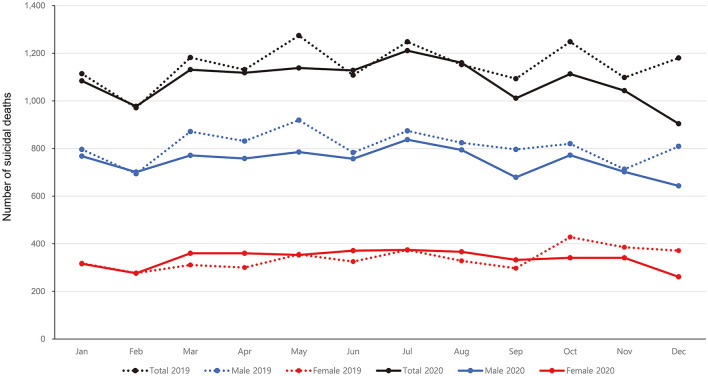
Number of suicidal deaths in South Korea (provisional values).

In contrast, the number of suicides seems to decrease in Korea (49). Using the monthly provisional number of suicides reported by Korean Statistical Information Service (30), we performed a chi-squared test to evaluate differences in the monthly distribution of suicides during the COVID-19 pandemic compared with the previous year (2019 vs. 2020). A highly significant difference in distribution of deaths by suicide (χ^2^ = 36.20, df = 11, *p* = 0.0002) was shown, which were found in both genders (Male: χ^2^ = 21.00, df = 11, *p* = 0.0335; Female: χ^2^ = 47.74, df = 11, *p* = 0.00002). Overall, the total number of suicides from January to December 2020 is 781 cases lower than that of the same period in the previous year. However, while males showed decreases in the number of suicides throughout the year, females showed slight increases in number in March, April, June, August, and September (Figure 2).

### Delayed Impacts of the COVID-19 - Honeymoon Phase

How can we explain the mismatch between the psychological response and the change in number of suicides? In the initial phase after a natural disaster, literature suggests there may be a brief decrease in suicide rates, a phase called the “honeymoon phase” (50, 51). There is much energy, optimism, and altruism that creates community bonding, accompanied by readily available assistance. This phenomenon has been observed in many national disasters including Hurricane Katrina in 2005 (52), the 9/11 terrorist attack in 2001 (53), the Great East Japan Earthquake in 2011(54), and the outbreak of Severe acute respiratory syndrome (SARS) in Hong Kong (55). However, as time goes on, the honeymoon phase comes to an end. The socioeconomic and psychological burdens remain, while the assistance may discontinue. In Japan, about 80% of the cash benefits (an amount of approximately $940) was distributed to all citizens before June and claims for business subsidies grew rapidly in the beginning of the epidemic, which may have contributed to the initial decline in suicide rates (45). Whereas, a downtrend has been observed in the suicide rates of many countries in the early phase of the pandemic, one cannot readily assume the upcoming picture to be optimistic.

### Long-Term Psychiatric Sequelae of the COVID-19

As coronavirus vaccines are being rolled out in many countries, the aftermath of the pandemic and what it represents for our mental health must be considered. While the infected cases are commonly accompanied by insomnia, anxiety, impaired cognitive function during the acute stage, they can extend to symptoms of post-traumatic stress disorder (PTSD), anxiety, and depression in the post-illness stage of coronavirus infection (56). Longer-term psychiatric sequelae remain unknown. The people closest to the infected cases—the families and frontline healthcare workers—have also experienced acute and post-traumatic stress (57). In the larger scope, no one could escape the stress and fear of getting infected or of having their loved ones infected. This generalized fear of illness and uncertainties contributed to the elevated anxiety and depression among the general population in many countries. How will this affect the future of mental health?

Social isolation, quarantine measures, and consequent deregulated emotions led to an increase in parental stress, children's psychological problems (58), and family violence (59, 60). Notably, adverse childhood events, such as childhood maltreatment, contribute to the development of psychiatric disorders, interfering with a child's brain development (61). Convincing evidence also suggests that pregnant women were more likely to experience anxiety and depression, and were more vulnerable to domestic violence during the pandemic (62, 63), while maternal mental illnesses have well-known adverse consequences for infant and child development (64). During the Spanish flu in 1918, the birth cohorts *in utero* displayed reduced educational attainment, increased rates of physical disability, lower income, and lower socioeconomic status in the USA (65). Similarly, children and adolescents today are also affected by the extreme social distancing measures such as school closures and lockdown restrictions. Besides the consequences on their immediate mental health (66), social restrictions can hamper the development of their social brain. Wearing masks also limits the children's experience of learning to read facial expressions, and the subtle nuances in language and communication (67). All this suggests the psychiatric sequelae of the COVID-19 can last for decades to come.

### Mental Health in the “New Normal”

The pandemic crisis brought an acceleration of the Fourth Industrial Revolution, transforming every facet of our society. While new technologies such as teleconferencing and the Internet of Things permeate our everyday lives nowadays, many remain unable to adapt to such rapid digitalization of social infrastructure, causing them anxiety (“techno-anxiety”) or aversion thereto – “techno-phobia.” At the opposite end, the extensive and compulsive use of internet and smartphones can cause “techno-addiction.” A systematic review by La Torre et al. suggests that information overload and its constant availability can cause a condition called “techno-stress,” characterized by higher circulating levels of cortisol, poor concentration, memory disturbances and irritability (68).

The COVID-19 pandemic has also created the need to implement and expand the use of remote communication technologies in health care. While traditional face-to-face interventions are disrupted and group therapies discontinued due to social distancing, telepsychiatry opens new opportunities for patients, allowing them to easily and safely access their mental health services (69, 70). Use of different technology platforms and settings such as applications can also be helpful for efficient monitoring and delivery of appropriate interventions (71). Although more evidence is required to examine the cost-effectiveness of digital psychiatry compared to the “in-person” care, the increase in demand for digital psychiatry is likely to improve its availability and quality in the market.

## Conclusion

To conclude, although the COVID-19 pandemic will be contained 1 day, its psychiatric impact can persist both as direct and indirect consequences of the viral infection and as responses to the “new normal.” Its effects can be generalized in every aspect of our society, manifesting themselves among adults, children, families, and in workplaces. Over the past century, globalization allowed infectious diseases to rapidly spread around the world. Severe Acute Respiratory Syndrome (SARS-CoV) and Middle East Respiratory Syndrome (MERS-CoV) were recent examples, but the COVID-19 pandemic is incomparable in terms of its extensiveness in time and place. As infectious epidemics can recur and their psychiatric implications will follow, it is crucial to expand our funding and international collaboration to understand the impact of epidemics on mental health.

## Data Availability Statement

The original contributions presented in the study are included in the article/Supplementary Material, further inquiries can be directed to the corresponding authors.

## Author Contributions

SM, JWK, and YA contributed to the literature review, data acquisition, analysis, and interpretation. The first draft of the manuscript was written by SM. YHJ, JK, JWK, and YA read and revised the manuscript and approved the final version. All authors conceptualized the manuscript.

## Funding

This work was supported by the KBRI basic research program (21-BR-02-13 and 21-BR-03-02 to YHJ, 21-BR-02-05 and 21-BR-03-04 to JK and 21-BR-02-06 and 21-BR-03-08 to JWK).

## Conflict of Interest

The authors declare that the research was conducted in the absence of any commercial or financial relationships that could be construed as a potential conflict of interest.

## Publisher's Note

All claims expressed in this article are solely those of the authors and do not necessarily represent those of their affiliated organizations, or those of the publisher, the editors and the reviewers. Any product that may be evaluated in this article, or claim that may be made by its manufacturer, is not guaranteed or endorsed by the publisher.
