# Supplementary material for: The Aftermath: Post-pandemic Psychiatric Implications of the COVID-19 Pandemic, a South Korean Perspective
Source: Front Psychiatry. 2021 Oct 21;12:671722. doi: 10.3389/fpsyt.2021.671722 (PMC8566744; doi:10.3389/fpsyt.2021.671722)
Supplement: Supplementary file 1 [file Table_1.pdf]

Supplementary table 1. Example of systematic review search strategy for PubMed

Search number	Query
15	#12 NOT #13 NOT #14
14	((((((((((((((media) OR HIV) OR cardiovascular) OR diabetes) OR pregnant) OR children) OR maternal) OR patients) OR students) OR facilities) OR nurse) OR nursing) OR pain) OR cancer) OR injury) OR veteran)
13	(((((Case Reports[Publication Type]) OR Guideline[Publication Type]) OR News[Publication Type]) OR Newspaper Article[Publication Type]) OR Review[Publication Type])
12	#5 AND #11
11	#6 OR #7 OR #8 OR #9 OR #10
10	((((Suicide) OR Attempted suicide) OR Suicide tendency) OR Suicide ideation) OR Self-harm
9	((((((((((Stress) OR Distress) OR Mental distress) OR Emotion distress) OR Stress-related disorder) OR Trauma-related disorder) OR Traumatic neurosis) OR Posttraumatic stress disorder) OR Posttraumatic symptom) OR PTSD) OR PTSS) OR Emotion trauma
8	((((Anxiety) OR Anxious) OR Generalized anxiety disorder) OR GAD) OR Social anxiety
7	((((Depression) OR Depressive) OR Depress) OR Depressive disorder) OR Depressive symptoms
6	((((((((Mental) OR Mental Health) OR Mental Disorder) OR Mental illness) OR Mental problems) OR Psychosocial) OR Psychosomatic) OR Unintended consequences) OR Common mental disorder
5	#1 AND #2 AND #3 AND #4
4	(((long-term) OR chronic) OR longitudinal)
3	(((Quarantine) OR Isolation) OR Confinement)
2	((((((((((Public) OR General public) OR General population) OR Citizens) OR People) OR Person) OR Community) OR Healthy) OR Quarantine) OR Isolation) OR Confinement
1	((((((((((((Coronavirus) OR Betacoronavirus) OR Coronavirus disease 2019) OR COVID-19) OR COVID) OR Novel coronavirus) OR 2019-nCoV) OR nCoV) OR Severe Acute Respiratory Syndrome) OR SARS) OR SARS-CoV-2) OR SARS-COV) OR Wuhan pneumonia
