# Supplementary material for: The Aftermath: Post-pandemic Psychiatric Implications of the COVID-19 Pandemic, a South Korean Perspective
Source: Front Psychiatry. 2021 Oct 21;12:671722. doi: 10.3389/fpsyt.2021.671722 (PMC8566744; doi:10.3389/fpsyt.2021.671722)
Supplement: Supplementary file 2 [file Table_2.pdf]

Supplementary table 2. Summary of included studies in the meta-analysis. *excluded in quantitative synthesis

Leading author	Year	Country	Period of data collecting (Data Source)	Sample size (Before vs. During)	Outcome reported	Assessment tool	Category of outcome	etc.
**Ettman**	2020	USA	2017 to 2018 vs. March 31, 2020 - April 13, 2020 (National Health & Nutrition Examination Survey)	5,065 vs. 1,441	Increase in depression prevalence (>3-fold)	PHQ-9 ≥ 10	Depression	
**Twenge**	2020	USA	January - June 2019 vs. April 23 - May 26, 2020 (National Health Interview Survey & Household Pulse Survey)	336,525	Increase in depressive disorders, anxiety disorders, or one or both (>3-fold)	PHQ-2 ≥ 3<br>GAD-2 ≥ 3	Depression<br>Anxiety	
**Castellini**	2020	Italy	December 1, 2019 - January 15, 2020 vs. April 22 - May 3, 2020	130	Increase in depressive symptoms, but no changes in anxiety	BSI	Depression<br>Anxiety	
***Ramiz**	2021	France	November 2014 and December 2019 vs. April 15, 2020 and May 4, 2020 (MAVIE cohort)	1,237	Depression remained unchanged from 27.0% at recruitment to 27.6% at lockdown. Anxiety symptoms increased from 17.3 to 20.1%	PHQ-9 > 4<br>GAD-7 > 4	Depression<br>Anxiety	No. with PHQ-9 ≥ 10 not indicated<br>No. with GAD-7 ≥ 10 not indicated
***van der Velden**	2020	Netherlands	March 2019 vs. March 2020 (Dutch longitudinal population-based LISS panel)	3,983	No increase in depression and anxiety symptoms	MHI-5 < 60	Depression<br>Anxiety	No value specific to depression and anxiety
***van der Velden**	2021	Netherlands	November 2019, March 2020, and June 2020 (Dutch longitudinal population-based LISS panel)	4,084	Lower prevalence of anxiety and depression after the outbreak (June 2 020, 15.3%) than before (16.8%) and during the outbreak (17.2%)	MHI-5 < 60	Depression<br>Anxiety	No value specific to depression and anxiety
**Winkler**	2020	Czech	November 2017 (Czech Mental Health Study) vs. May 6 - 20, 2020	3,306 vs. 3,021	Increase in depression (3-fold) and anxiety symptoms (2-fold)	MINI	Depression<br>Anxiety	
**Brailovskaia**	2020	Germany	October 2019 vs. March 20, 2020 (Bochum Optimism and Mental Health project)	436	Depression and anxiety at baseline (October 2019) did not predict burden at follow-up (March 2020). But, stress symptoms assessed at baseline were a significant predictor of higher burden at follow-up	DASS-21	Depression<br>Anxiety<br>Stress	
**Ruggieri**	2020	Italy	March 7 - 9, 2020 (pre-quarantine)<br>March 25 - 27, 2020 (post-quarantine)<br>April 12 - 14, 2020 (post-quarantine)	March 7 - 9, 2020 : 113<br>March 25 - 27, 2020 : 75<br>April 12 - 14, 2020 : 80	Increase in depression, anxiety, and stress	DASS-21	Depression<br>Anxiety<br>Stress	
**Haliwa**	2021	USA	September - December 2019 vs. April - June 2020<br>Sample 1 : December 4 - 10, 2019 vs. April 3 - 15, 2020<br>Sample 2 : October 6 - 13, 2019 vs. May 14 - 25, 2020<br>Sample 3 : September 18 - 26, 2019 vs. June 10 - 19, 2020<br>(Amazon's Mechanical Turk)	Sample 1 : 300<br>Sample 2 : 146<br>Sample 3 : 142	Significant increases in anxiety and stress.<br>But no change in depression	Depression :<br>- Sample 1 & 3 : PHQ-8 ≥ 10<br>- Sample 2 : DASS-21<br>Anxiety :<br>- Sample 1 & 3 : GAD-7 ≥ 10<br>- Sample 2 : DASS-21<br>Stress :<br>- Sample 1 & 3 : PSS<br>- Sample 2 : DASS-21	Depression<br>Anxiety<br>Stress	Analysis with sample 1 and sample 3 for depression and anxiety; sample 2 for psychological distress
**Ayuso-Mateos**	2021	Spain	June 17, 2019 - March 14, 2020<br>May 21, 2020 - June 30, 2020 (Edad con Salud project)	1,103	No change in depression and suicidal ideation	CIDI 3.0	Depression<br>Suicidal ideation	
***Sueki and Ueda**	2021	Japan	January 24, 2020 vs. April 27 and 30, 2020	6,683	Decrease in suicidal ideation	Suicidal ideation scale	Suicidal ideation	
***Savolainen**	2021	Finland	September 16 and October 15, 2019 vs. September 15 and October 22, 2020 (Social Media at Work in Finland survey)	1,044	Increase in psychological distress<br>Women and young people experienced higher anxiety	STAI-6<br>GHQ-12	Psychological distress<br>Anxiety	Not suitable measure in anxiety (post-pandemic anxiety as dependent variable). No. with psychological distress not indicated (GHQ values not given)
**McGinty**	2020	USA	2018 (National Health Interview Survey) vs. April 7 - 13, 2020 (Johns Hopkins COVID-19 Civic Life & Public Health Survey)	25,417 vs. 1,468	Increase in psychological distress	Kessler 6 ≥ 13	Psychological distress	
**Pierce**	2020	UK	2018-19 vs. April 2020 (UK household longitudinal study)	17,452	Increase in psychological distress	GHQ-12 ≥ 4	Psychological distress	
**Niedzwiedz**	2020	UK	2017–2019 vs. April 23-30, 2020 (UK household longitudinal study)	9,748	Increase in psychological distress	GHQ-12 ≥ 4	Psychological distress	
**Ferry**	2020	UK	January 2017- December 2018 vs. April 2020 (UK household longitudinal study)	8,708	Increase in psychological distress	GHQ-12 ≥ 4	Psychological distress	
**Canady**	2021	USA	February 2019 vs. May 2020 (Rand American Life Panel)	2,555 vs. 1,870	No change in psychological distress	Kessler 6	Psychological distress	
**Kuhn**	2021	Switzerland	September 2, 2019 - March 3, 2020 vs. May 12 - June 30, 2020 (Swiss Household Panel)	5,859	Reduced stress levels significantly	Stress was captured with the question how often respondents felt stressed during the last 2 weeks, with answers ranging from never (1) to very often (5)	Perceived stress	
**Novotny**	2020	Czech	December 19, 2014 vs. April 24 to May 27, 2020	715	Increase in stress (1.4-fold) and depressive symptoms (5.5-fold)	PSS<br>PHQ-9 (before COVID-19)<br>+ PHQ-4 (during COVID-19) ≥ 3	Depression<br>Perceived stress	No. with PHQ-9 ≥ 10 not indicated<br>No. with GAD-7 ≥ 10 not indicated
***Paschke**	2021	Germany	September 13 - 27, 2019 vs. April 20 - 30, 2020	824 adolescents<br>824 respective parents	Increase in psychological stress during COVID-19	PSS-4 ≥ 8	Perceived Stress	No. with psychological distress not indicated (PSS values not given)
***Daly**	2020	UK	2017–2019 vs. April, May, and June 2020 (UK household longitudinal study)	14,393	Prevalence of mental health problems (GHQ-12 ≥ 3) increased from 24.3% in 2017-2019 to 37.8% in April 2020 and remained elevated in May (34.7%) and June (31.9%) 2020	GHQ-12 ≥ 3	Psychological distress	Same sample as Niedzwiedz et al., 2020
