# Supplementary material for: The Aftermath: Post-pandemic Psychiatric Implications of the COVID-19 Pandemic, a South Korean Perspective
Source: Front Psychiatry. 2021 Oct 21;12:671722. doi: 10.3389/fpsyt.2021.671722 (PMC8566744; doi:10.3389/fpsyt.2021.671722)
Supplement: Supplementary file 3 [file Data_Sheet_1.docx]

**Supplementary materials**

**Methods for the systematic review and meta-analysis**

**Study selection and data extraction**

A systematic search was conducted for longitudinal studies that measured changes in mental health of the general population since the COVID-19 pandemic that were published from January 1, 2020 to July 12, 2021. Electronic searches using subject headings (i.e. MeSH terms) and free-text keywords (shown in Supplementary table 1) involved five electronic databases: PubMed, Scopus, Web of Science, APA PsychInfo, and CINAHL. According to the indices of each database, key search terms used for mental health included “mental health”, “mental illness”, “mental disorder”, “depression”, “anxiety”, “stress”, “post-traumatic stress disorder” and “suicide”. Key search terms used for COVID-19 included “coronavirus disease 2019”, “novel coronavirus”, and “SARS-CoV-2”. Manual search using the reference lists of the selected papers was done to identify additional potential target studies. The study population was limited to the general population, without age restriction (See Supplementary figure 1). While we performed the electronic searches, we found a study that analyzed real-time suicide rates from 21 countries (31). As all data on suicide rates from the selected studies from our search were included in Pirkis et al., we determined another systematic review on the same topic to be redundant and therefore excluded “suicide” from our systematic review.

Three authors independently screened the titles and abstracts, and reviewed the full text articles to select studies meeting the following criteria: studies (a) with longitudinal designs (i.e., cohort studies or repeated cross-sectional studies); (b) that assessed psychological symptoms before and during the COVID-19 pandemic (the reference date March 11, 2020 being the day the World Health Organization declared a pandemic) using the same measurement tools; (c) that are validated and standardized (e.g., the Patient Health Questionnaire-9 (PHQ-9) for depression, the Generalized Anxiety Disorder-7 (GAD-7) for anxiety, etc.). We excluded studies with retrospective design and studies that focused on specific populations such as health care workers, COVID-19 survivors, or college students. Unpublished articles (i.e., not peer-reviewed), non-English articles, commentaries, case series, case studies, and reviews were excluded. No studies were excluded based on sample size and study duration.

JWK extracted the primary data from the eligible studies; the extracted data were independently verified by SM. The following data were extracted from each selected study: title, first author name, year of publication, study location(s), sample size, study design, period of data collecting, measurement tools used, outcome measure, and main results (Supplementary table 2). We did not ask authors for additional data that were not reported in the studies, neither were they found in their supplementary materials.

**Statistical analysis**

Quantitative synthesis was done separately for depression, anxiety, and psychological distress. We calculated and transformed effect sizes to log odds ratios following the guidelines of Borenstein 2021 (32). A random-effects meta-analysis using restricted maximum likelihood as a heterogeneity variance estimator was conducted. The *I^2^* statistic and Cochran’s *Q* test were used to assess the statistical heterogeneity. Qualitative and quantitative assessments of publication bias were performed using funnel plots and Egger’s regression tests, respectively. Sensitivity analysis was conducted to evaluate the impact of differences in measurement tools on pooled results and to explore potential sources of heterogeneity. In addition, chi-squared test was conducted to assess differences in the monthly distribution of male, female, and total suicides in South Korea during the COVID-19 pandemic compared with the previous year (2019 vs. 2020). All statistical tests were two-tailed and p<0.05 was considered statistically significant. All statistical analyses were performed using R software (version 4.1.0; R development Core Team, Vienna, Austria) and the R *metafor* package (version 3.0-2).
