# Supplementary figures and images for: The Aftermath: Post-pandemic Psychiatric Implications of the COVID-19 Pandemic, a South Korean Perspective
Source: Front Psychiatry. 2021 Oct 21;12:671722. doi: 10.3389/fpsyt.2021.671722 (PMC8566744; doi:10.3389/fpsyt.2021.671722)

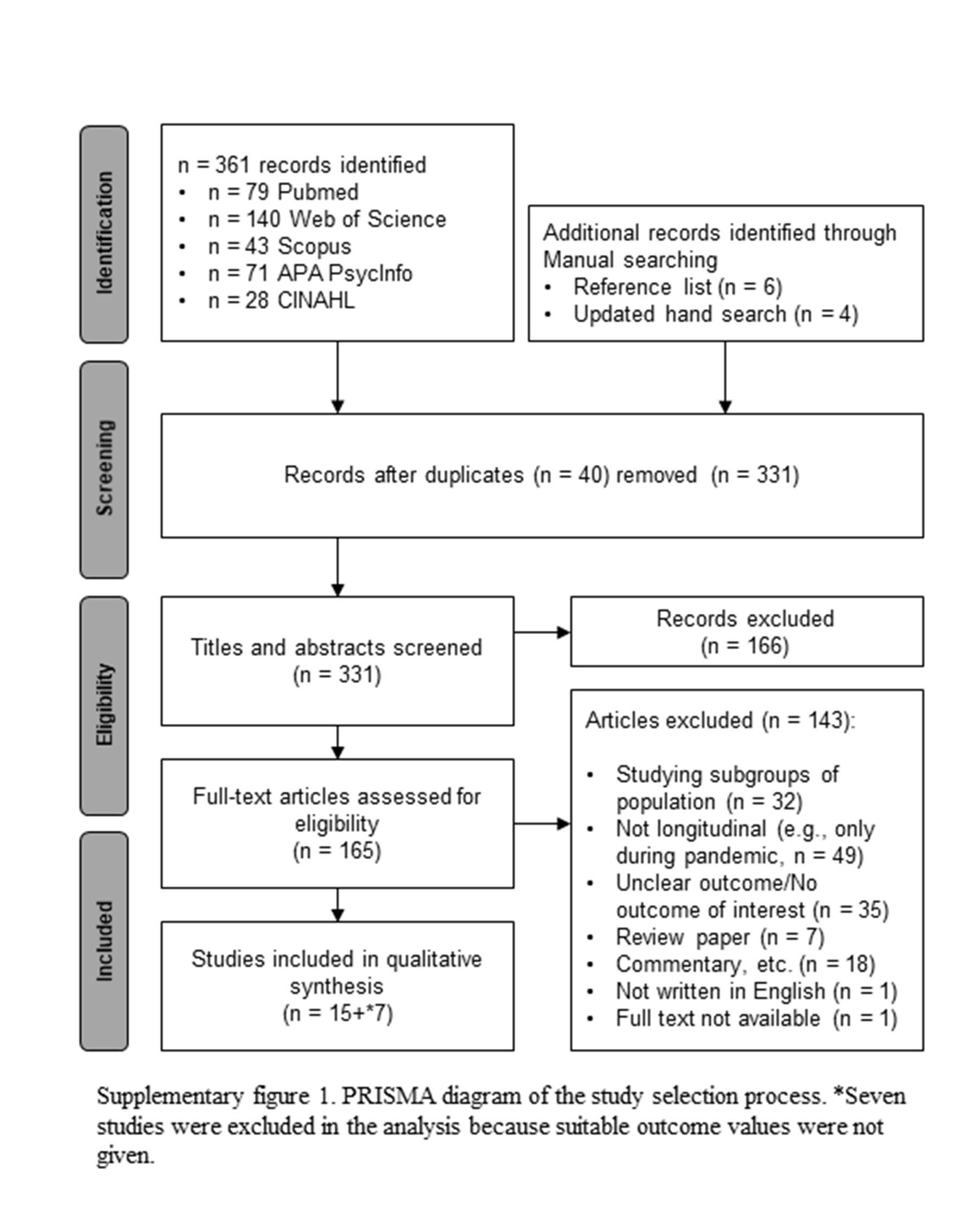

Supplement: Supplementary file 4 [file Image_1.TIF]

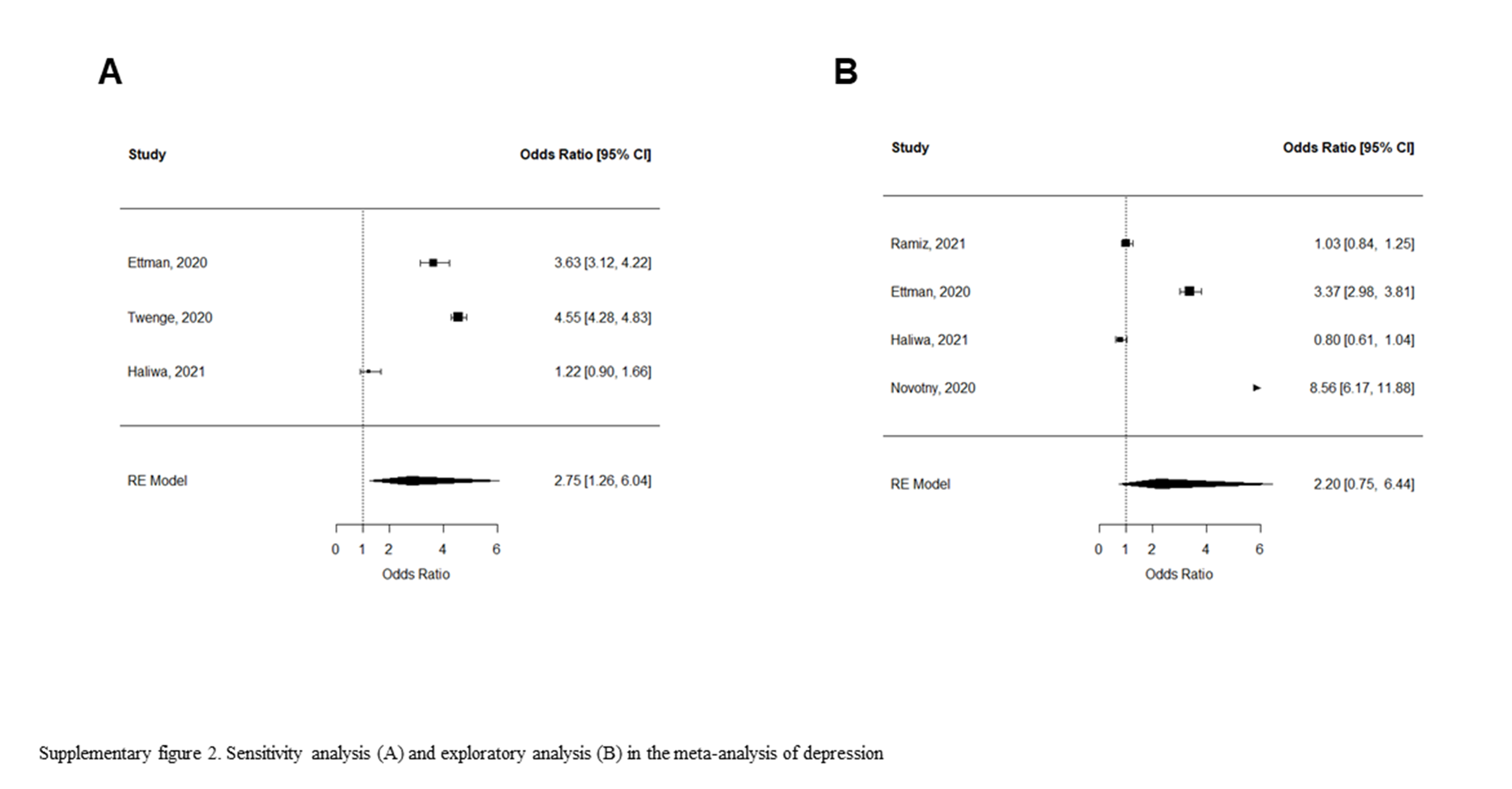

Supplement: Supplementary file 5 [file Image_2.TIF]
